# Patterns of handgun divestment among handgun owners in California

**DOI:** 10.1186/s40621-021-00362-6

**Published:** 2022-01-03

**Authors:** Sonja A. Swanson, Matthew Miller, Yifan Zhang, Lea Prince, Erin E. Holsinger, Zachary Templeton, David M. Studdert

**Affiliations:** 1 Department of Epidemiology, Erasmus University Medical Center, P.O. Box 2040, 3000 CA Rotterdam, The Netherlands; 2 Department of Health Sciences, Northeastern University, Boston, MA USA; 3 Stanford Center for Health Policy, Stanford University, Stanford, CA USA; 4 Department of Healthcare Management and Economics, University of Pennsylvania, Philadelphia, PA USA; 5 Stanford Law School, Stanford University, Stanford, CA USA

**Keywords:** Firearm, Handgun, Divestment

## Abstract

**Background:**

Little is known about voluntary divestment of firearms among US firearm owners. Here, we aim to estimate the proportion of handgun owners who divest their handguns in the years following their initial acquisition; examine the timing, duration, and dynamics of those divestments; and describe characteristics of those who divest.

**Methods:**

We use data from the Longitudinal Study of Handgun Ownership and Transfer, a cohort of registered voters in California with detailed information on 626,756 adults who became handgun owners during the 12-year study period, 2004–2016. For the current study, persons were followed from the time of their initial handgun acquisition until divestment, loss to follow-up, death, or the end of the study period. We describe the cumulative proportion who divest overall and by personal and area-level characteristics. We also estimate the proportion who reacquired handguns among persons who divested.

**Results:**

Overall, 4.5% (95% CI 4.5–4.6) of handgun owners divested within 5 years of their first acquisition, with divestment relatively more common among women and among younger adults. Among those who divested, 36.6% (95% CI 35.8–37.5) reacquired a handgun within 5 years.

**Conclusions:**

Handgun divestment is rare, with the vast majority of new handgun owners retaining them for years.

**Supplementary Information:**

The online version contains supplementary material available at 10.1186/s40621-021-00362-6.

## Key messages

What is already known:

- One in five US adults owns a firearm.
- The only prior study of firearm divestment estimated 2% of US adults change from gun owners to former gun owners over a 5-year period.

What this study adds:

- In our study of registered voters in California who purchased a handgun, handgun divestment was rare with nearly 95% of handgun purchasers remaining owners for 5 or more years.
- Among those who divested, one-third reacquired a handgun within 5 years of divestment.

## Introduction

Firearm ownership is prevalent in the USA, with an estimated 300 million firearms distributed such that one in five US adults owns a firearm and one in three households contains a firearm (Smith and Son 2015; Azrael et al. 2015; Smith et al. 2018). Although these proportions have been relatively stable for more than two decades, few studies have used individual-level data to examine how personal firearm ownership changes over time. In particular, little is known about shifts from personal ownership to non-ownership. To our knowledge, only one study published in the peer review literature has investigated such shifts empirically: In a cross-sectional survey that assessed past firearm ownership retrospectively, Wertz et al. estimated that approximately 2% of US adults change from gun owners to former gun owners in a 5-year period (Wertz et al. 2019). The paucity of information about patterns and predictors of divestment hampers initiatives that aim to voluntarily reduce exposure to guns as a means of reducing injury.

The Longitudinal Study of Handgun Ownership and Transfers (LongSHOT) (Zhang et al. 2020) follows a cohort of approximately 28 million registered voters in California over a 12-year period (2004–2016) and includes detailed information on 626,756 adults who became handgun owners for the first time during this period. We used the LongSHOT cohort to estimate the proportion of handgun owners who divest their handguns after an initial acquisition, describe the timing and durability of divestment, and characterize divesters.

## Methods

### Study design

The study was approved by the institutional review board at Stanford University. Details of LongSHOT have been described at length elsewhere (Zhang et al. 2020; Studdert et al. 2020). In brief, LongSHOT was formed by linking information on handgun transfers and all-cause mortality among adults in California to a series of 13 historical extracts of the California Statewide Voter Registration Database (SVRD). The extracts were spaced approximately 1 year apart, on average, and spanned the period October 18, 2004, through May 23, 2016. The SVRD includes all registered voters in the state—approximately 61% of all adult residents—creating a large sample of adults known to be alive and residing in California.

Virtually, all lawful transfers of firearms in California must be transacted through a licensed firearms dealer (California Penal Code 2012). This includes persons who move to California with firearms, as they are required to report their weapons within 60 days of arrival. Dealers must relay details of the transfers to the California Department of Justice (CADOJ), which archives this information in the Dealer Record of Sale (DROS) database. This has been done with handgun transfers for decades, and for long gun transfers since 2014. We linked handgun transfers archived in the DROS database between January 1, 1985, and February 29, 2016, to the SVRD files. This historic database on handgun transfers allowed us to identify handgun acquisitions, de-acquisitions, and divestment. For the current study, we restricted the cohort to persons who did not own a handgun when they entered LongSHOT. This restriction meant our analysis focused on divestment patterns among people who acquired their first handgun ever (or at least since January 1, 1985) between October 18, 2004, and February 29, 2016, with follow-up beginning at the time of an individual’s first acquisition.

We define divestment as cessation of ownership of all handguns a person owns, where such de-acquisitions may occur through selling, gifting, or otherwise no longer legally owning the handgun. The exact date in which a person purchases a handgun can be obtained from DROS transfer records. The de-acquisition of a handgun is identified by observing the unique weapon identifier appearing in a subsequent transfer. Here, divestment was defined as occurring on the date of the transfer. As described in prior LongSHOT studies (Zhang et al. 2020; Studdert et al. 2020), this definition of divestment timing accurately reflects the date of ownership cessation for private party transfers but is the latest possible date consistent with other transfer types (e.g., dealer sale, pawn redemption by a new individual, curio/relic registration).

### Measurement of demographic and area-level characteristics

Handgun owners were characterized at the time of their first acquisition by personal and area-level characteristics. We used information from the SVRD extracts to identify their sex (male, female, unknown/other) and age (categorized here as 21–34, 35–49, 50–64, and 65 years and older). Race/ethnicity categories (Asian, non-Hispanic Black, Hispanic, other, unknown, non-Hispanic White) were imputed by a procedure developed and validated by Imai and Khanna (Khanna 2019; Imai and Khanna 2016), which uses information on census block, surname, sex, and birthdate.

Because almost nothing is known about the characteristics of gun owners who subsequently divest of their firearms, we describe not only demographic characteristics but also area-level factors that may be related to the primary reasons people offer for acquiring firearms: protecting oneself, one’s family, and one’s property (Azrael et al. 2015). We created four area-level variables based upon the residential address of each person as specified in the SVRD extract just prior to their acquisition: socioeconomic status index; total violent and property crime rates; and urbanicity. The Agency for Healthcare Research and Quality socioeconomic status index was computed based on calendar year and census tract (Bonito et al. 2008; Krieger et al. 2003; Lang et al. 2008). Index values were categorized by quartiles. Total violent and property crime rates (per 10,000 persons per year) were estimated at the county and calendar year level. Numerators for the crime rates came from the official state statistics collected by the Criminal Justice Statistics Center (https://openjustice.doj.ca.gov/data). We computed rates of violent crimes (homicides, rapes, robberies, and aggravated assaults) and property crimes (burglaries, motor vehicle thefts, and larceny thefts) separately. Denominators for these rates came from official population estimates for each of the 58 counties in each of the study years (State of California DoF 2011, 2019). Both types of crime rates were categorized by quartiles. Urbanicity was categorized according to the Rural Urban Commuting Area (RUCA) codes classification system developed by the federal government (United States Department of Agriculture Economic Research Service 2020) and used the aggregation scheme recommended by the Washington State Department of Public Health (Hailu and Wassermanh 2016). We linked RUCA codes from the 2010 Census to individuals’ residential census tract.

### Analyses

We estimated the cumulative proportion who divested over time following their initial handgun acquisition. Persons were followed from the time of their initial handgun acquisition until divestment, loss to follow-up (defined as the date of the first SVRD extract in which they did not appear), or February 29, 2016 (whichever came first). Death during follow-up was treated as a competing event such that a person who died while still owning a handgun by definition never divested. Cumulative proportions were further estimated by subgroups defined by the measures described above. Among persons who divested, we computed the cumulative proportion who reacquired handgun(s) following initial divestment and tabulated patterns in subsequent divestment and reacquisitions. All cumulative proportions and their corresponding 95% confidence intervals (CIs) were estimated using a Kaplan–Meier estimator. Analyses were completed using R version 3.4.2.

## Results

**Fig. 1:**
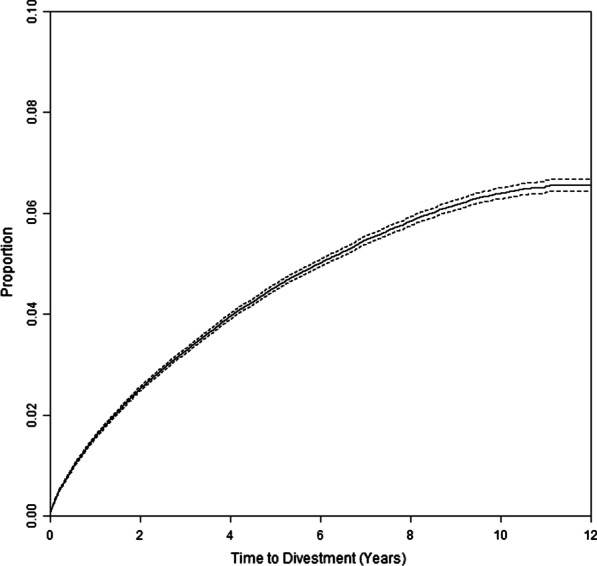
Time to handgun divestment among first-time acquirers of handguns

Of the 626,756 persons who acquired their first handgun during the study period, 78.5% were men (Table 1). A total of 22,279 divested during follow-up. Median follow-up time was 3.2 years. Of those who were not observed to divest, 9,429 died during follow-up, 86,683 were lost to follow-up, and 508,365 remained in the study until the end of the study period. Overall, the estimated percent who divested within 1, 2, 5, and 10 years was 1.6% (95% CI 1.5–1.6), 2.5% (95% CI 2.5–2.6), 4.5% (95% CI 4.5–4.6), and 6.4% (95% CI 6.3–6.5), respectively (Fig. 1).

**Table 1:** 1-year and 5-year divestment by personal and area-level characteristics at time of first handgun acquisition

	N	%	Proportion divesting by 1 year, % (95% CI)	Proportion divesting by 5 years, % (95% CI)
Overall	626,756		1.6 (1.5, 1.6)	4.5 (4.5, 4.6)
Sex*				
Male	492,234	78.5	1.5 (1.4, 1.5)	4.3 (4.3, 4.4)
Female	133,577	21.3	1.9 (1.8, 2.0)	5.3 (5.1, 5.4)
**Racial or ethnic group**				
Non-Hispanic White	470,686	75.1	1.4 (1.4, 1.5)	4.1 (4.0, 4.2)
Hispanic	98,082	15.6	2.0 (1.9, 2.1)	6.1 (5.9, 6.3)
Asian	27,866	4.4	1.9 (1.7, 2.1)	5.2 (4.8, 5.5)
Non-Hispanic Black	26,017	4.2	2.1 (1.9, 2.3)	5.9 (5.5, 6.3)
Other	991	0.2	1.8 (0.9, 2.6)	6.5 (4.5, 8.5)
**Age, years**				
21–34	254,451	40.6	2.0 (1.9, 2.0)	6.0 (5.9, 6.1)
35–49	171,364	27.3	1.4 (1.3, 1.4)	4.0 (3.9, 4.1)
50–64	148,987	23.8	1.2 (1.1, 1.2)	3.1 (3.0, 3.2)
65 +	51,954	8.3	1.4 (1.3, 1.5)	3.5 (3.3, 3.7)
**Area socioeconomic status**				
Quartile 1	156,690	25.0	1.9 (1.8, 1.9)	5.5 (5.4, 5.7)
Quartile 2	156,688	25.0	1.5 (1.4, 1.6)	4.6 (4.4, 4.7)
Quartile 3	156,700	25.0	1.4 (1.3, 1.5)	4.2 (4.1, 4.3)
Quartile 4	156,678	25.0	1.5 (1.4, 1.5)	3.9 (3.8, 4.1)
**County total violent crime rate**				
Quartile 1	157,113	25.1	1.5 (1.5, 1.6)	4.3 (4.2, 4.5)
Quartile 2	156,471	25.0	1.5 (1.4, 1.5)	4.4 (4.2, 4.5)
Quartile 3	157,415	25.1	1.6 (1.6, 1.7)	4.8 (4.7, 4.9)
Quartile 4	155,757	24.9	1.6 (1.6, 1.7)	4.6 (4.5, 4.8)
**County total property crime rate**				
Quartile 1	160,204	25.6	1.4 (1.4, 1.5)	4.3 (4.1, 4.4)
Quartile 2	154,771	24.7	1.6 (1.6, 1.7)	4.5 (4.4, 4.7)
Quartile 3	155,964	24.9	1.6 (1.5, 1.7)	4.6 (4.5, 4.7)
Quartile 4	155,817	24.9	2.6 (2.5, 2.7)	4.7 (4.6, 4.8)
Urbanicity*				
Urban core	517,704	82.6	1.6 (1.6, 1.6)	4.6 (4.6, 4.7)
Suburban	73,899	11.8	1.3 (1.3, 1.4)	4.0 (3.8, 4.2)
Large rural town	20,263	3.2	1.6 (1.4, 1.7)	4.4 (4.1, 4.8)
Small town isolated rural	14,888	2.4	1.1 (0.9, 1.3)	3.5 (3.1, 3.9)

*There are 945, 3114, and 2 individuals with unknown sex, race/ethnicity, or urbanicity, respectively

Divestment patterns differed by personal and area-level characteristics (Table 1; Additional file 1: Supplemental Figs. 1–7). Women were more likely than men to divest throughout follow-up. For example, women were about 20% more likely than men to divest within 5 years, with 5.3% (95% CI 5.1–5.4) compared to 4.3% (95% CI 4.3–4.4) divesting by then. Younger adults (aged 21–34 years at the time of acquisition) were most likely to divest throughout follow-up. For example, they were nearly twice as likely to divest within 5 years as were those who acquired a handgun when they were 50–64 years old (6.0% [95% CI 5.9–6.1] vs. 3.1% [95% CI 3.0–3.2]). Throughout follow-up, divestment was least likely among Non-Hispanic Whites compared to other race/ethnicity groups and was more likely among people who lived in areas that were socioeconomically disadvantaged when they acquired a handgun. Urbanicity and total property or violent crime rates of the area a person lived at the time they acquired a handgun were less consistent of predictors of subsequent divestment.

**Fig. 2:**
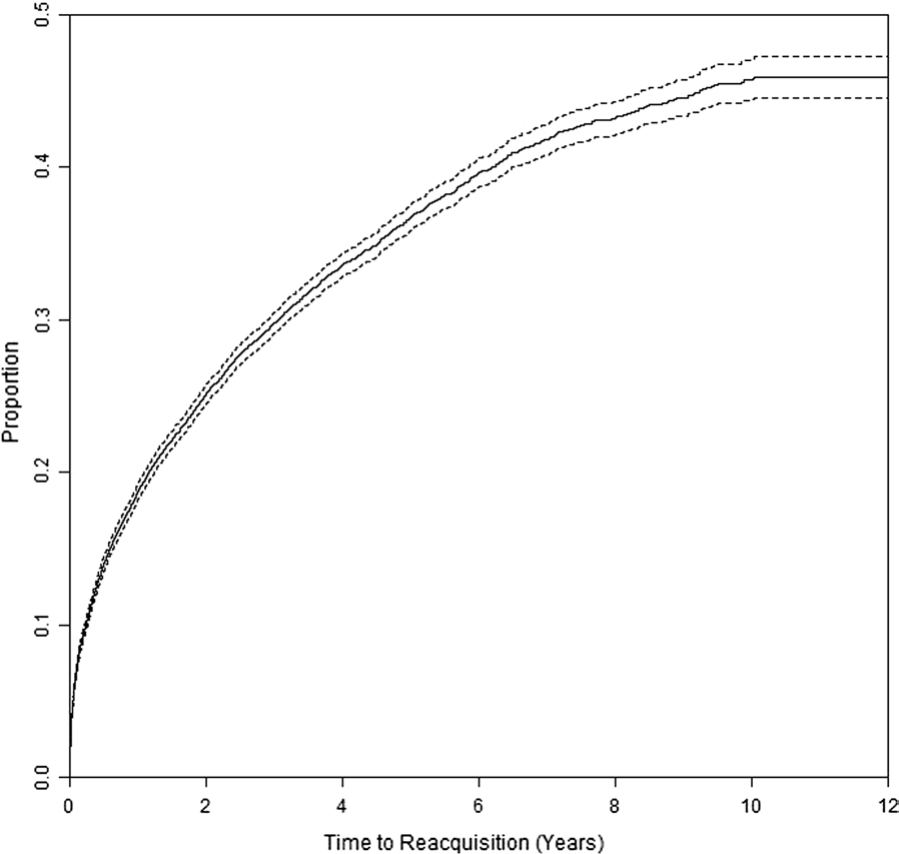
Time to reacquisition among persons who had divested their handguns

Multiple handguns were owned by 2,456 (11.0%) divesters at some point between their first acquisition and first divestment. Among divestments defined by a single final transfer (N = 21,532; as opposed to multiple handguns being transferred), most transaction types were private party transfers (N = 12,729, 59.1%) and dealer sale (N = 6,271, 29.1%); the remainder consisted of a variety of smaller transaction types (e.g., curio/relic sales, pawn redemptions) that are categorized separately by CADOJ. A total of 6,397 of the divesters were observed to reacquire one or more handguns. One-third of divesters (36.6% [95% CI 35.8–37.5]) reacquired handguns within 5 years of divestment (Fig. 2).

Subsequent divestments and reacquisitions within the study period were relatively rare: Among divesters, 714 (3.2%) were observed to have reacquired and divested exactly once more, 93 (0.4%) did so exactly twice more, and 26 (0.1%) did so three or more additional times.

## Discussion

Divestment was relatively rare, with the vast majority of new handgun owners remaining owners throughout follow-up. Moreover, one-third of divesters reacquired handguns within 5 years. Divestment was somewhat more common among women, among younger adults, and among those living in more socioeconomically disadvantaged areas at the time of their initial handgun acquisition.

Our estimates complement the only previous assessment of divestment incidence and extend those findings by providing novel information on characteristics of divesters and the durability of divestment. Wertz et al. estimated that 2% of all US adults and therefore about 10% of US gun owners changed from gun owners to former gun owners over a 5-year period (4). The difference between that estimate and ours (4.5%) may be explained by differences in study design (e.g., self-report vs. centrally tracked transfers; retrospective vs. prospective data collection); differences in study populations (e.g., nationally representative vs. Californian registered voters; all gun owners vs. new handgun owners); random error; and definition of divestment (all firearms vs. handguns only).

Our findings should be viewed with several considerations in mind. First, our definition of handgun acquisition, ownership, and divestment, at best, captures only lawful transfers since 1985. But handgun owners may part ways with their handguns in a variety of ways, including theft, loss, or unlawful transfer. Moreover, defining divestment based on the date of transaction to a new owner means that for some types of transfers (e.g., dealer sales), the true timing of the divestment may have been weeks or months earlier than the date recorded and available in these data. Second, we focus on divestment of handguns, regardless of long gun ownership, and on individual divestment, regardless of whether others in the household may own handguns. Third, our area-level characteristics are based on the residential location of handgun owners at the time of the SVRD extract just prior to acquiring their first handgun, not the time of divestment, and thus will not reflect the location of those who moved in the interim. Fourth, any patterns observed may not generalize to understanding divestment after a longer period of ownership or beyond Californian registered voters. For example, the background checks required in California for private sales of handguns may in fact alter the practice of lawful divestment compared to states without this requirement. Finally, a richer understanding of why handgun owners divest, including their personal motivations, the context in which they are motivated to divest, and the ways in which the individual characteristics studied here may intersect and interact, is an area for further research.

Several public health strategies for reducing firearm violence rely on voluntary divestment, including buyback programs and lethal means counselling. Results from our study may help to inform which types of gun owners will be more responsive to these initiatives. The fact that divestment was more common among younger adults and women might suggest that some life events (e.g., having a child) motivate divestment, or that members of these groups have weaker cultural attachments to their handguns. The regional patterns across socioeconomic status suggest that financial considerations may motivate some divestments. Several other public health strategies for reducing firearm violence focus on reducing new ownership or encouraging safer firearm storage practices. Here, too, our findings shed some light: Given that over 95% of new handgun owners continue to be owners for years afterward (and over one-third reacquire firearms within 5 years of initial divestment), injury prevention strategies implemented at the time of acquisition that encourage sustained safer storage practices could have an enduring effect (e.g., on youth suicide) if successful.

## Conclusions

In sum, findings from our prospective study add to a nearly nonexistent empirical literature regarding people who decide to cease being firearm owners. Future research should aim to better understand the motivations for divestment and explore effects of voluntary divestment on the risk of injury—both to former owners and to other members of former owners’ household. Together with descriptive information from studies like ours, such evidence would help design and tailor population-based initiatives aimed at reducing firearm violence.﻿

## Supplementary Information

**Additional file 1.** Supplementary materials on time to divestment by subgroups.

## Data Availability

The study data were formed by linking confidential datasets held by three state agencies in California (Department of Justice, Secretary of State, Department of Public Health). The three agencies have prescribed processes for making their data available to researchers.
