# Supplementary material for: Patterns of handgun divestment among handgun owners in California
Source: Inj Epidemiol. 2022 Jan 3;9:2. doi: 10.1186/s40621-021-00362-6 (PMC8725449; doi:10.1186/s40621-021-00362-6)

Online Supplement to "Patterns of handgun divestment among handgun owners in California"

Supplementary Figure 1. Time to divestment by sex at the time of initial handgun acquisition.

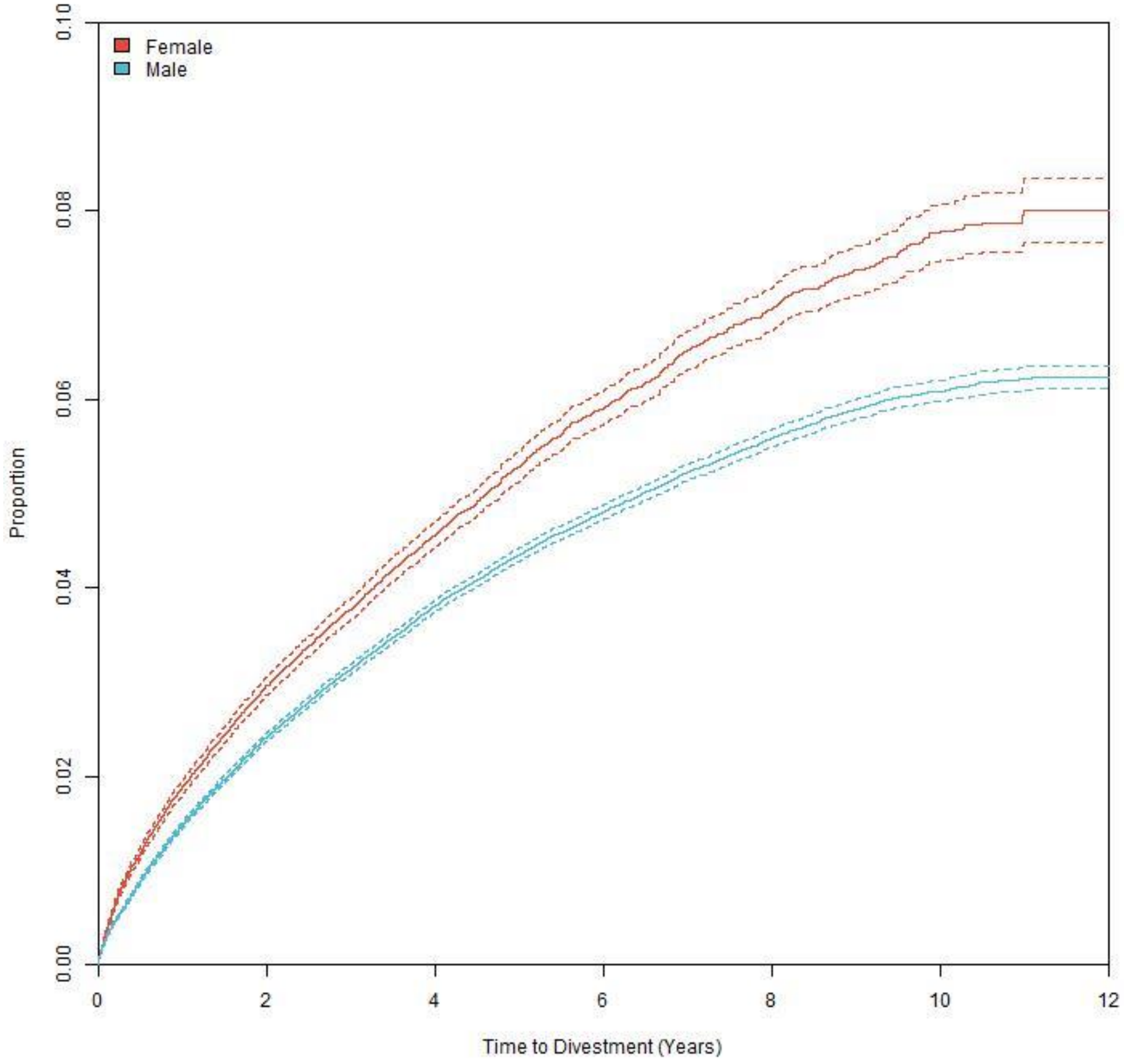

Supplementary Figure 2. Time to divestment by race/ethnicity at the time of initial handgun acquisition.

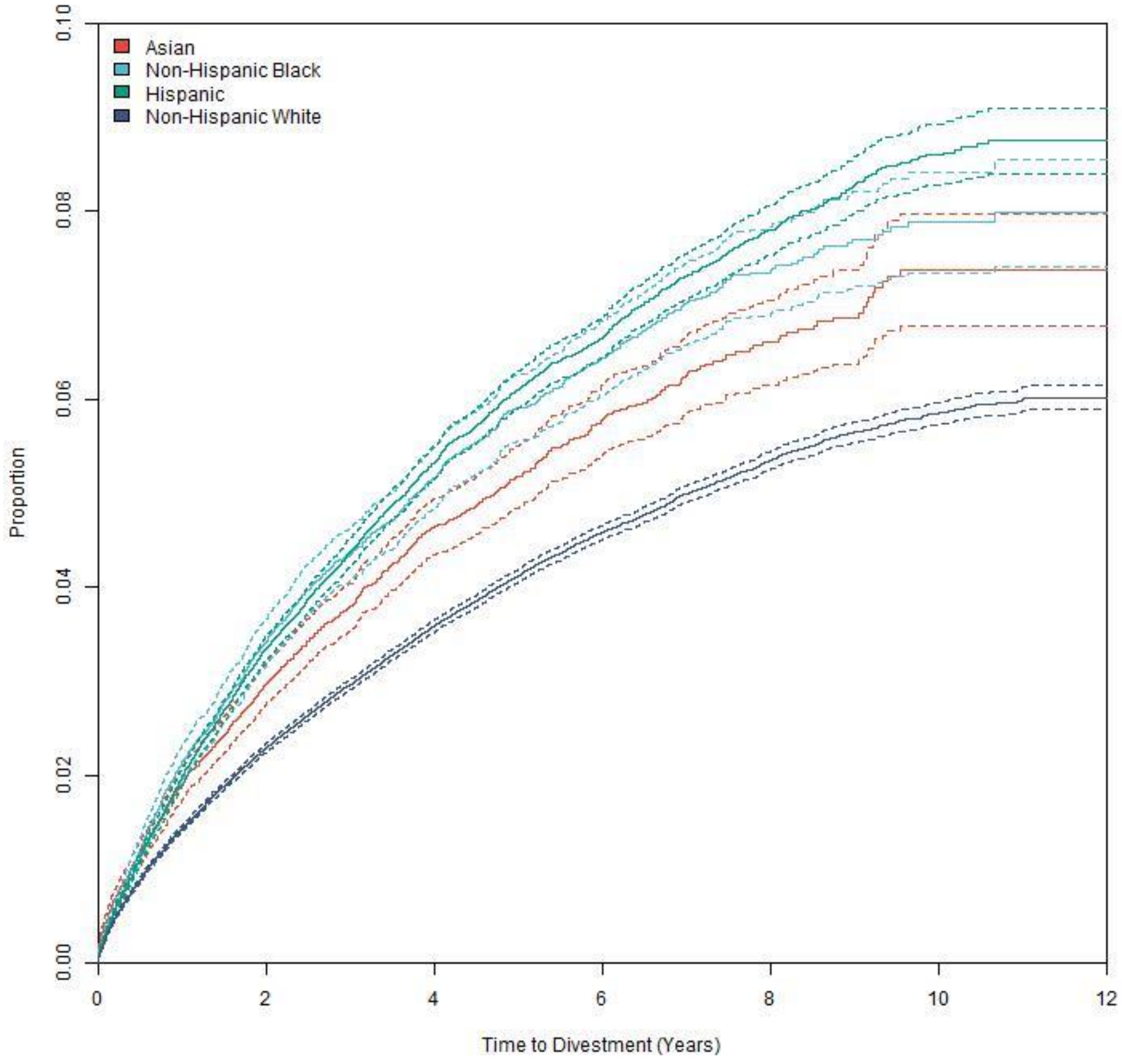

Supplementary Figure 3. Time to divestment by age group at the time of initial handgun acquisition.

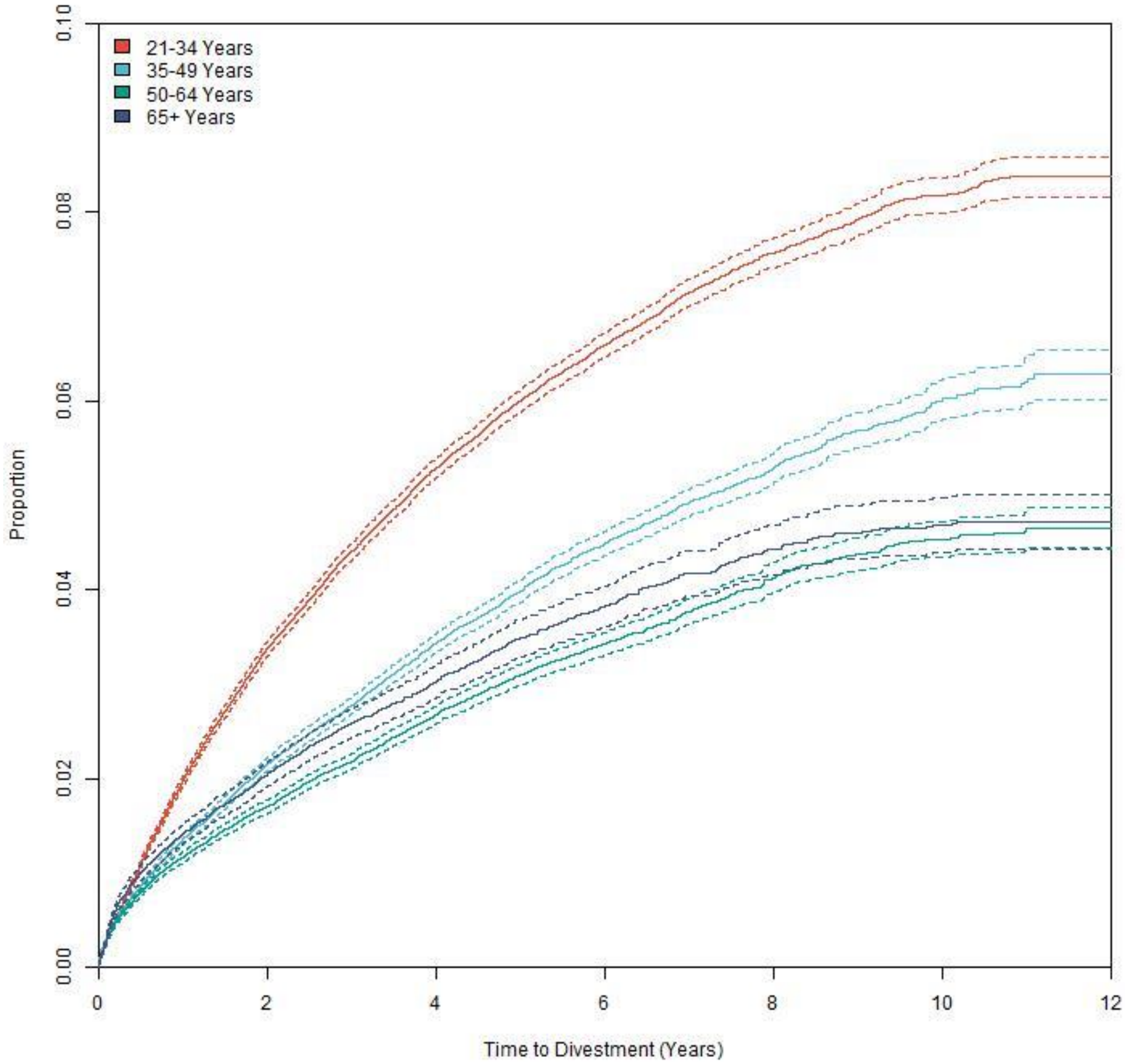

Supplementary Figure 4. Time to divestment by area-level socioeconomic status at the time of initial handgun acquisition.

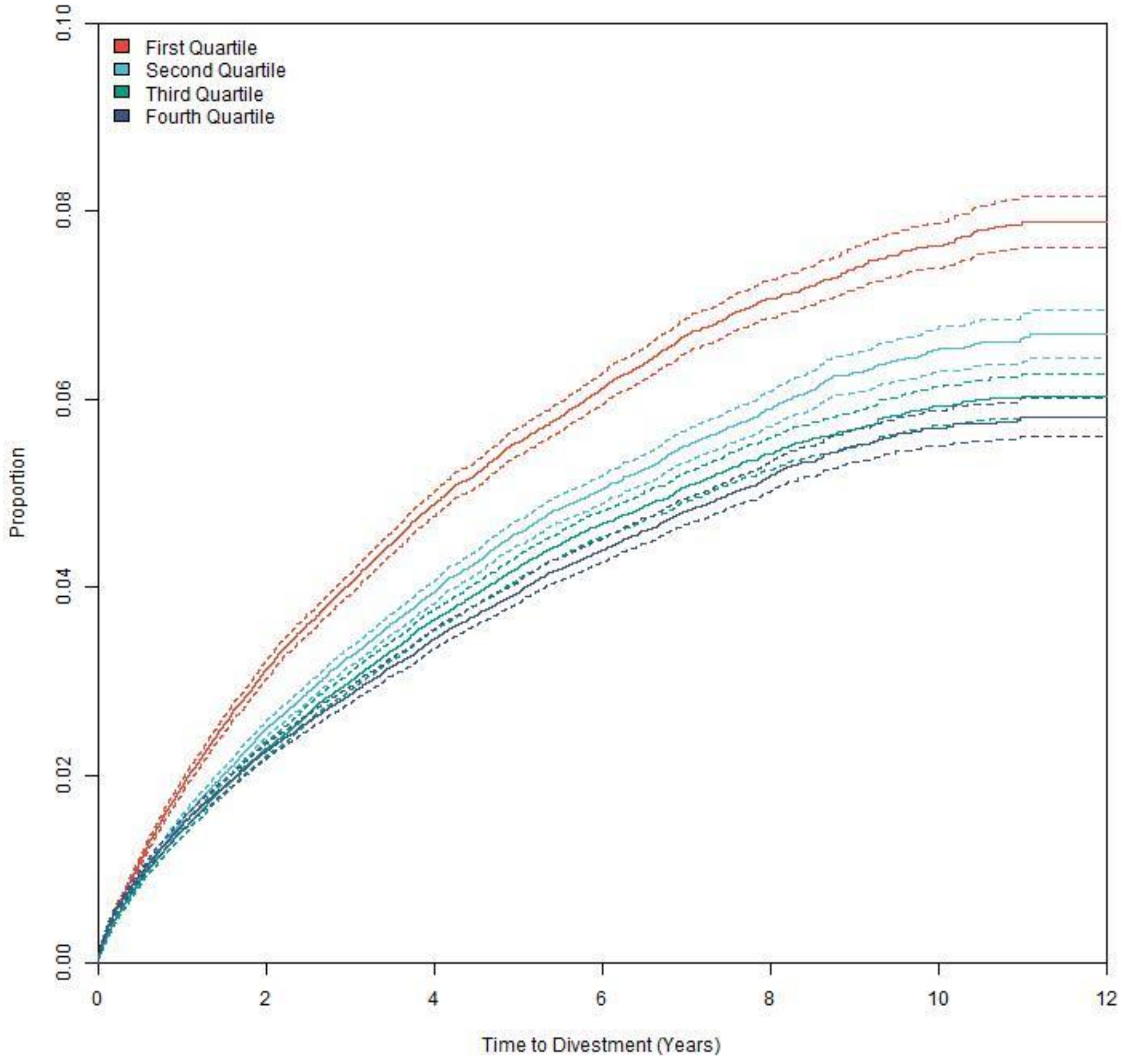

Supplementary Figure 5. Time to divestment by area-level total violent crime rate at the time of initial handgun acquisition.

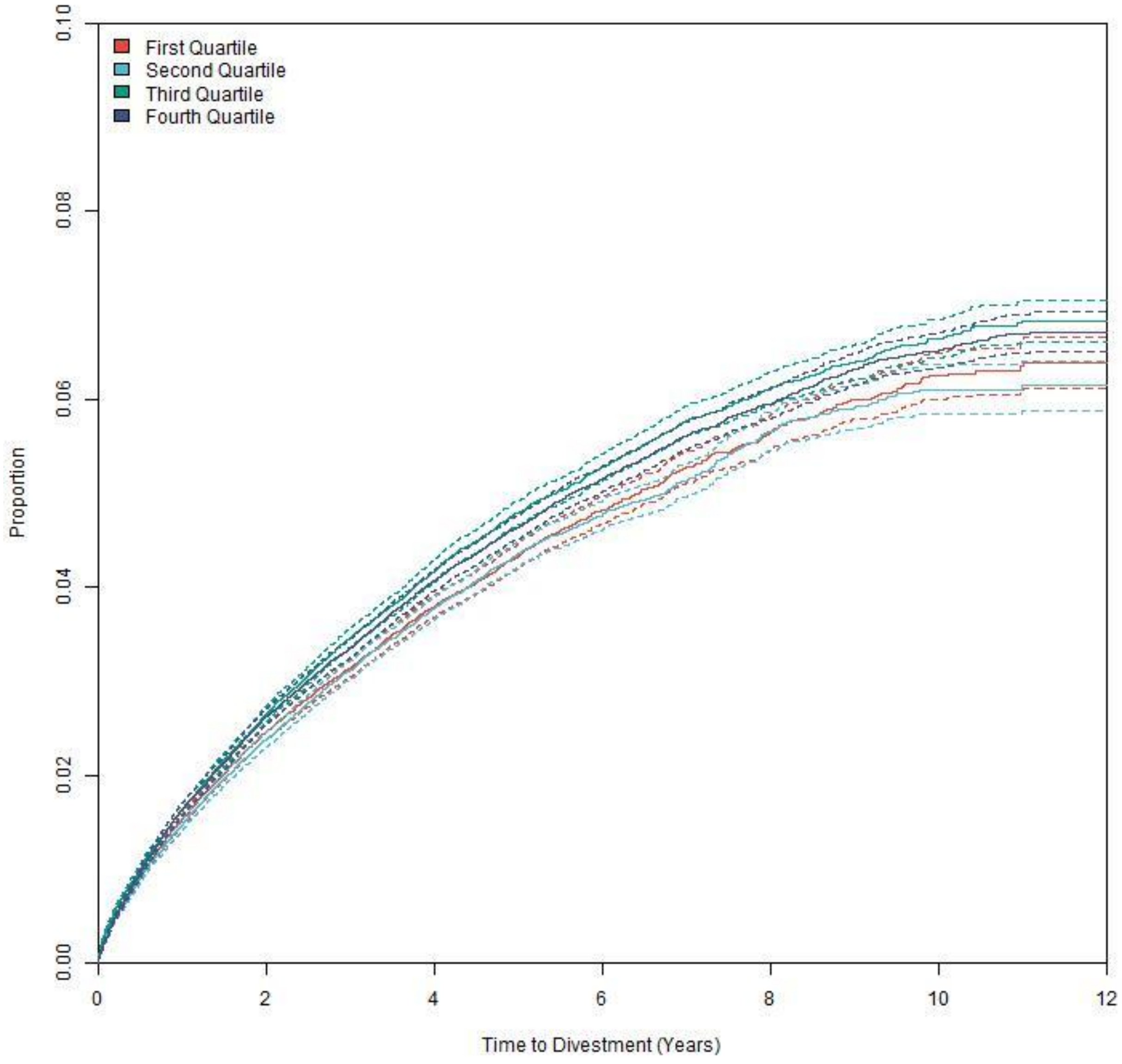

Supplementary Figure 6. Time to divestment by area-level total property crime rate at the time of initial handgun acquisition.

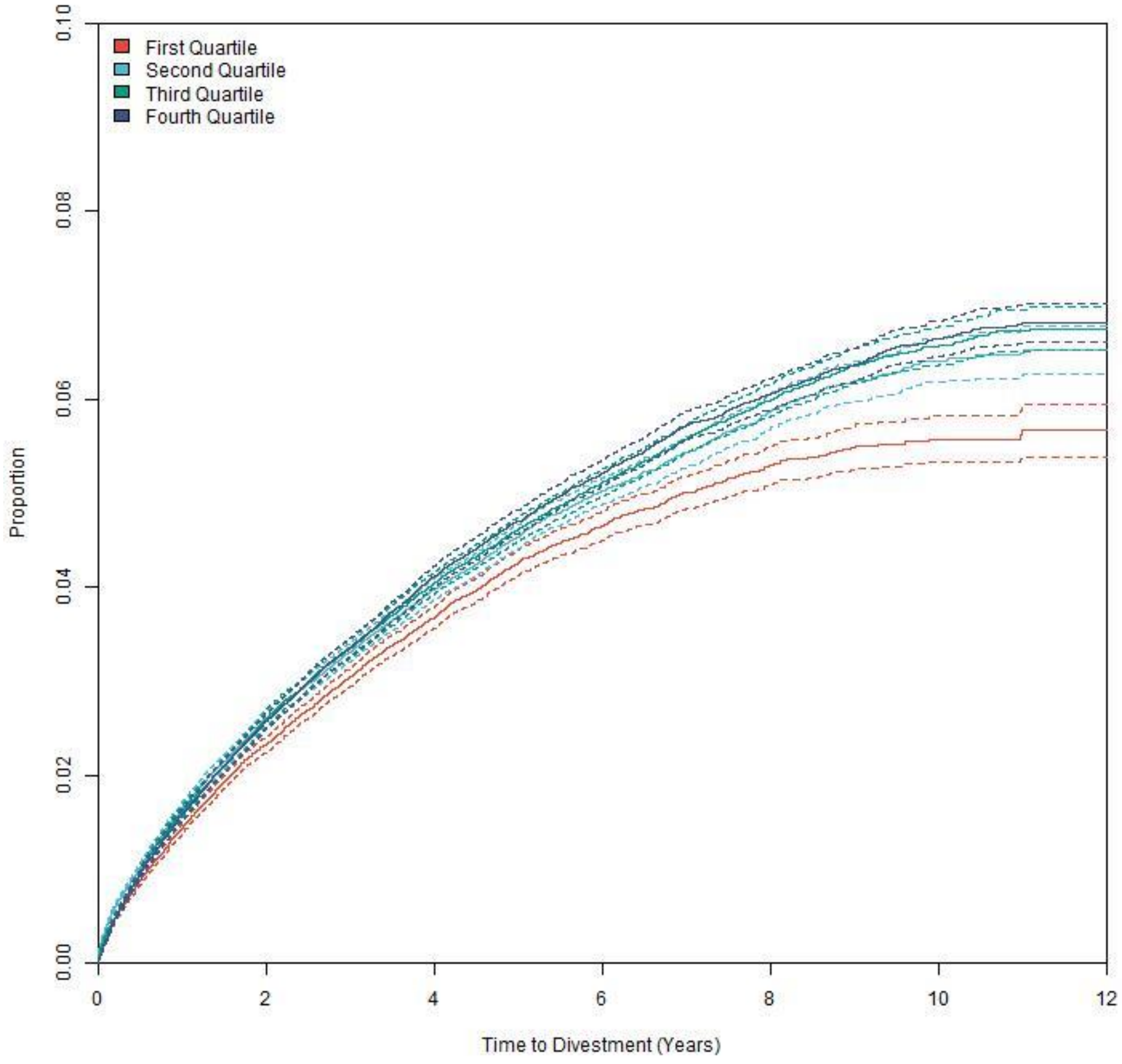

Supplementary Figure 7. Time to divestment by urbanicity at the time of initial handgun acquisition.

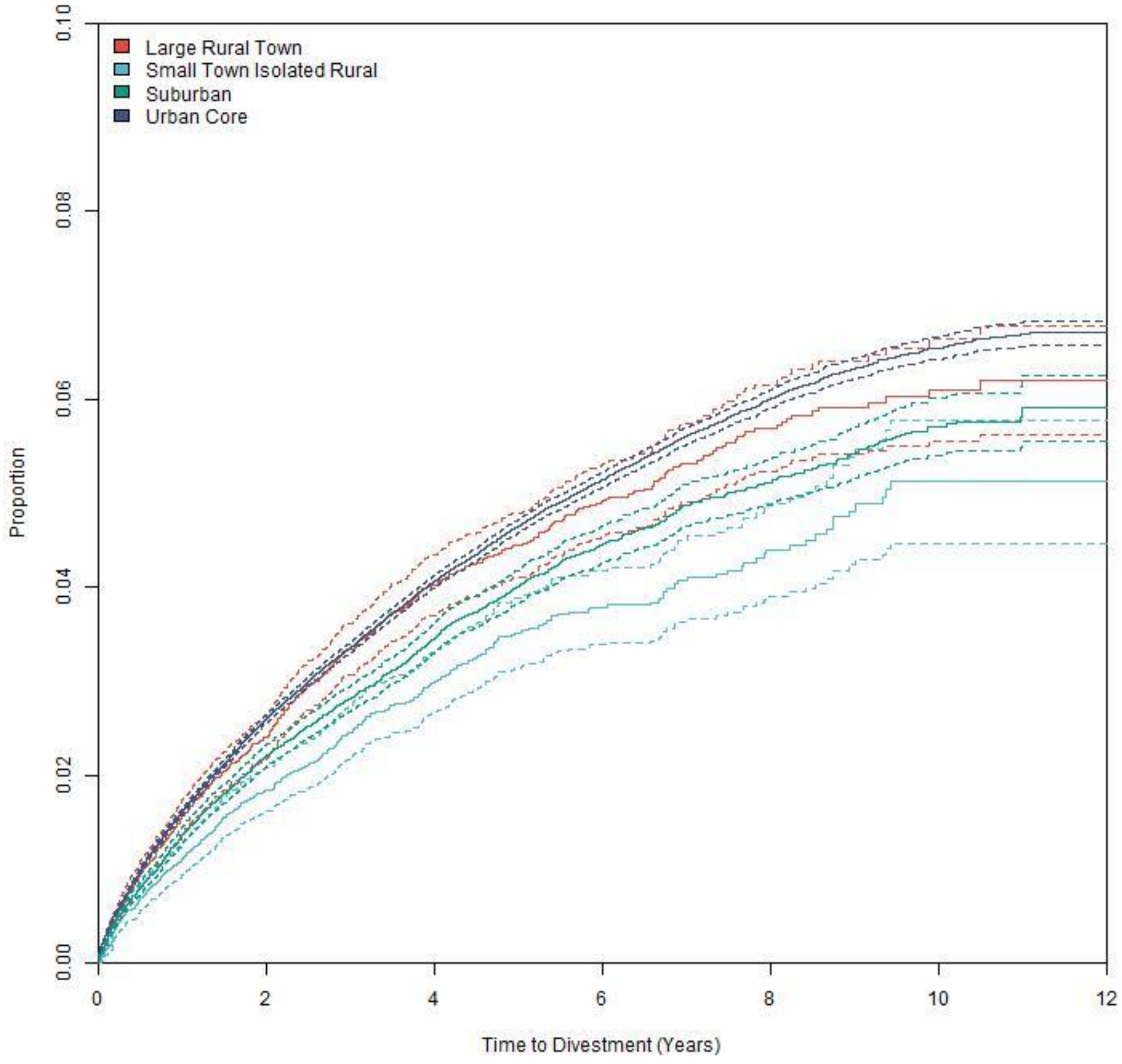

Supplement: Supplementary file 1 — Additional file 1. Supplementary materials on time to divestment by subgroups. [file 40621_2021_362_MOESM1_ESM.pdf]
